# Te-Pei Feng: a pioneer of neuromuscular physiology in China

**DOI:** 10.1007/s13238-021-00850-x

**Published:** 2021-06-07

**Authors:** Wei Xie, Ben-Yu Guo, Yanyan Qian

**Affiliations:** 1 School of Psychology, Nanjing Normal University, Nanjing, 210097 China; 2 Social and Behavioral Sciences Faculty, Leiden University, 2333AC Leiden, The Netherlands

**Figure 1:**
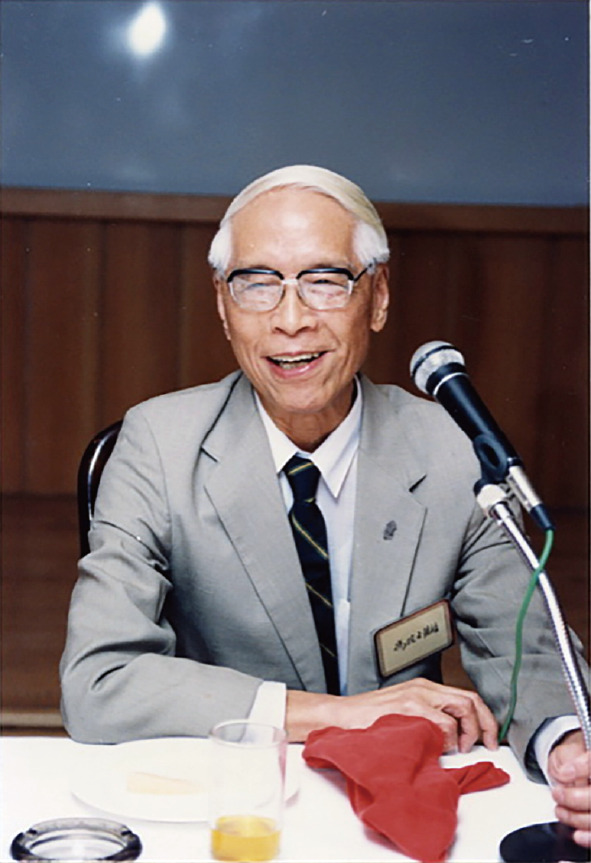
**Te-Pei Feng**

Te-Pei Feng (冯德培, 1907–1995), was a famous Chinese neurophysiologist and also one of the pioneers of modern physiological researches in China (Fig. 1). He mainly studied chemical transmission at the neuromuscular junction, the biological characteristic of high and low contraction speed muscles, and the long-term potentiation (LTP) function of the brain (Jin, 2007). Richard Wuyin Tsien (钱永佑) of Stanford University highly valued Feng’s contributions to neurology in China, stating that, “It is safe to say that the past and the future of neuroscience in China would be very different had professor Feng not provided leadership, by dint of organization, persuasion and strong personal example. Moreover, Feng’s remarkable life and career spanned a lengthy epoch of history, in both the world and scientific arenas, of scope unlikely to be matched again.” (Tsien, 2007). Feng’s student Gong Chen (陈功) said that Feng left three legacies: a. Feng’s scientific achievements, especially in the field of physiology; b. His leading role in establishing the Shanghai Institute of Physiology and promoting the development of Chinese physiological sciences; c. His spirit of never bending on the way of seeking truth (Chen, 2007).

Feng’s scientific achievements have laid the foundation for muscle neurology research in China, while the study of synaptic plasticity in nerve-muscle transmission mechanisms remains at the forefront of current research. At the Chinese Congress on Artificial Intelligence (CCAI) in 2018, Mu-Ming Poo (蒲慕明), member of the Chinese Academy of Sciences, prominently mentioned the phenomenon of synaptic plasticity, which was first discovered by Feng in 1941 (Feng, 1941; Poo, 2010). Much later, in 1973, Timothy Vivian Pelham Bliss and Terje Lømo discovered a similar phenomenon in the central nervous system (Bliss & Lømo, 1973). Even as late as 2020, Feng’s article published in *Chinese Physiology Journal* (*English edition*) in 1940 was still cited in a paper related to synaptic plasticity research (Ge, Noakes, & Lavidis, 2020). These two examples demonstrate the great enduring significance of Feng’s pioneering work in the field of synaptic plasticity.

**Figure 2:**
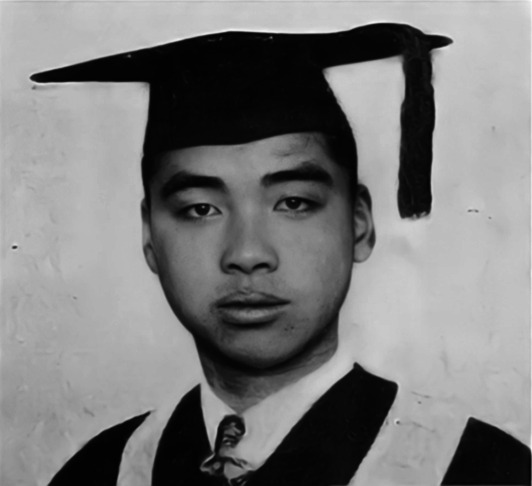
**Te-Pei Feng’s graduation photo from Fudan University in 1926** (Photo credit: http://www.zhongdangwang.com/)

Feng was born to a rich family in Linhai county of Zhejiang province in 1907. At only 11 years of age, he demonstrated excellent scholastic achievements and skipped grade to the Sixth Middle School of Zhejiang province. In 1922, Feng enrolled at the Department of Literature of Fudan University. The following year, Feng’s scientific interest was piqued by a science course given by the psychologist Zing-Yang Kuo (郭任远), who had returned to China from the United States (Qian, 1981; Feng, 1986). Then, Feng transferred to the Department of Psychology from the Literature Department of Fudan. In his junior year of university, Feng became the formal student of Professor Chiao Tsai (蔡翘), studying physiology, after which he obtained a bachelor’s in biology in 1926 from Fudan University (Fig. 2). After that, Feng also has been the lecturer recruited by Chiao Tsai at Fudan University for one year (1926–1927) (The Editorial Department of *World Science*, 1985). In the autumn of 1927, Feng was recommended by Tsai to the laboratory of Robert Kho-Seng Lim (林可胜) at the Department of Physiology of Peking Union Medical College (PUMC). There, he worked under the guidance of Lim, who studied the nervous and humoral control of gastric secretion.

As early as 1929, Te-Pei Feng made a pioneering discovery on the physiology of the alimentary canal, revealing the enterogastrone hormones of the stomach, for which he coined the term (Feng et al., 1929; Ji, 2007). During his time at the PUMC, Feng read extensively about almost all fields of physiology. Additionally, Feng also mastered 5 languages, including English, German, French, Spanish and Italian (Feng, 1986).

In the summer of 1929, Feng obtained Tsinghua University Fellowship to study in the United States. Because of his interest in Ralph Lillie’s book *Protoplasmic Action and Nervous Action*, he finally went to Chicago University and studied general physiology under biologist Ralph Stayner Lillie’s guidance. At that time, Lillie was preoccupied with the iron-wire model of nerve conduction, but what Feng really wanted to explore was real nerves, and not the model. Therefore, he quickly made a choice to go to another laboratory, which was led by neurophysiologist and behavioral scientist Ralph Waldo Gerard, who finally became Feng’s first tutor in neurophysiological research. Working with Gerard, Te-Pei Feng was mainly preoccupied with the mechanism of nerve damage due to asphyxiation. He investigated whether an asphyxiated nerve could be induced to recover by soaking it in an oxygen-free solution of certain oxidizing dyes like methylene blue, instead of giving it oxygen? Feng quickly discovered that the connective tissue sheath surrounding the nerve was an effective diffusion barrier, which could prevent the methylene blue from reaching the nerve fibers (Feng & Gerard, 1930). 

After earning his master’s degree from Chicago University in the autumn of 1930, Feng was recommended by Lim of PUMC to work with Hill at London University. There, Feng mainly worked on heat production, first in muscle and later in nerves. In the next two and a half years, Feng either conducted or participated in sufficient work for 9 papers, 5 of which he wrote up himself. In 1932 he discovered the increase of the resting heat production of muscle on passive stretch, which was called “stretch response” (Feng, 1932a). Later, muscle chemist Jakub Karol Parnas from Poland and the whole international physiology circle named it the “Feng Effect” (Wei, 1988). After that, Hill requested Feng to look into Lapicque’s controversial theory of isochronism of muscle and nerve under different conditions. Although he was convinced that Lapicque’s theory is incorrect according to the experimental results, he felt what he had done was not meaningful. Therefore, Feng decided to shift his research direction to the role of lactic acid in nervous activities. The same year, he discovered another new phenomenon, showing that lactic acid enables nerves to perform normal continuous function for many hours. Later, this discovery was described in the paper “*The role of lactic acid in nerve activity*” (Feng, 1932b).

After Feng got his doctoral degree in Physiology from the University College of London in 1933, he was recommended by Archibald Vivian Hill to stay in the laboratories of Edgar Douglas Adrian at Cambridge University and Charles Scott Sherrington at Oxford University for two months each, so that Feng could expand his study experience (Adrian and Sherington shared the Nobel Prize in Physiology or Medicine in 1932). Feng quickly learned about the advanced issues in 1930s that was which the information delivered in the neuromuscular junction is electrical transfer or electro-chemical transfer in neurophysiology? (Editorial Department of *World Science*, 1985). Although Feng worked in the Laboratory of Adrian for merely two months, he solved a longstanding problem of Adrian that was related to the relationship between potassium and the recovery of injured nerves. Later, Feng’s conclusion was verified repeatedly by Adrian and finally accepted (Feng, 1933). Before returning to China, Feng was recommended by Hill again to go back to the United States, where he spent one year working at the newly established Johnson Foundation for Medical Physics in Philadelphia, directed by Detlev Bronk. During that period, Feng devoted the majority of his time to learning how to assemble electronic instruments in preparation for establishing a laboratory in China.

**Figure 3:**
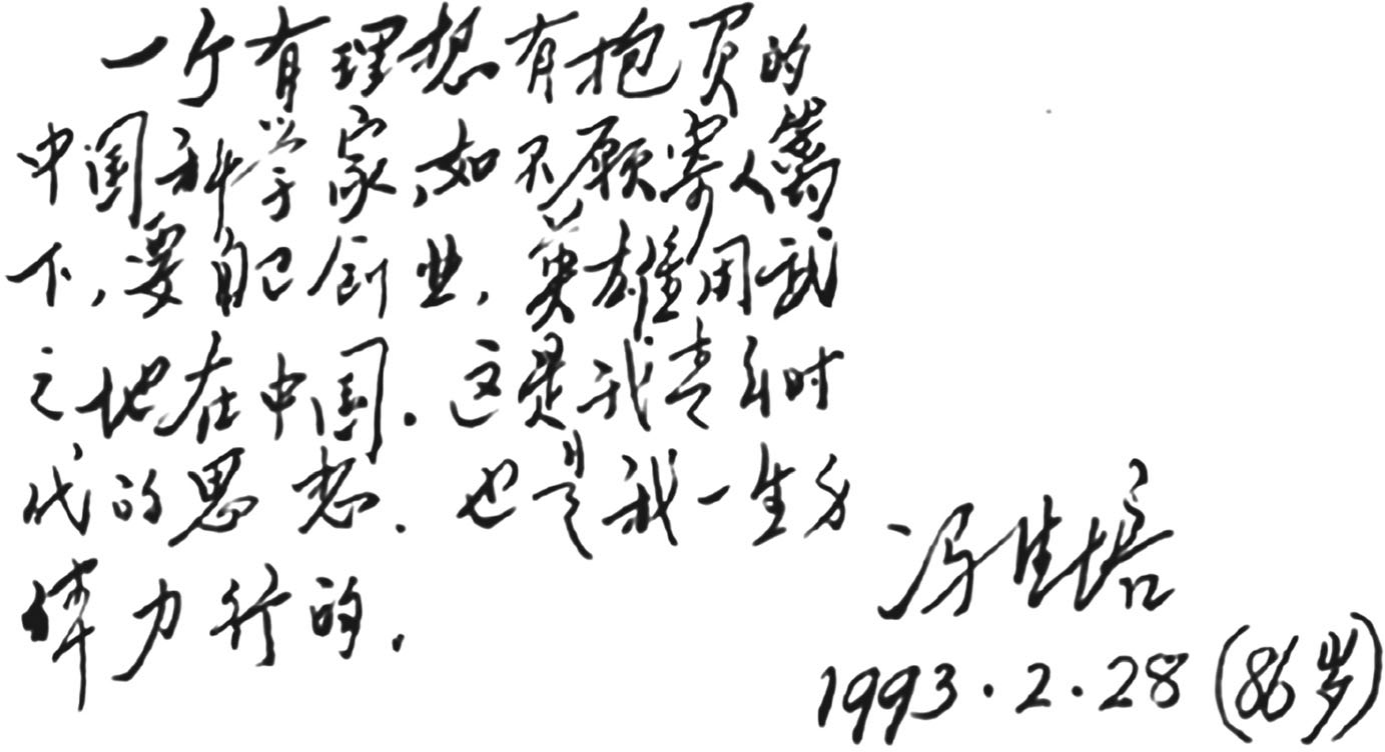
**Te-Pei Feng’s view on life’s purpose and message to young scholars** (Photo credit: Academician Library of Chinese Academy of Sciences https://yswk.csdl.ac.cn/)

With the idealism of the young adult period (Fig. 3), Feng returned to China in the summer of 1934 and set about establishing his laboratory. Because of the difficult socioeconomic circumstances at the time, the laboratory was set up in the basement. In spite of the hardship, Feng immediately started his experiments on nerves and muscles. Afterwards, Feng came to an important conclusion that the contraction elicited by direct muscle stimulation could be inhibited by additional nerve stimulation at certain frequencies. Due to this bout of intuition, Feng switched his research topic to the neuromuscular junction, developing the Nobel prize-level theory that calcium affects the release of neurotransmitters (Sa, 2015). Due to his previous achievements, and with recommendation of Hill, in 1936, Feng wrote a summary article “*The heat production of nerves*”, which was regarded by academic circles as an authoritative reference in the field of neural heat study and was published in the journal *Ergebnisse der Physiologie* (Feng, 1936).

**Figure 4:**
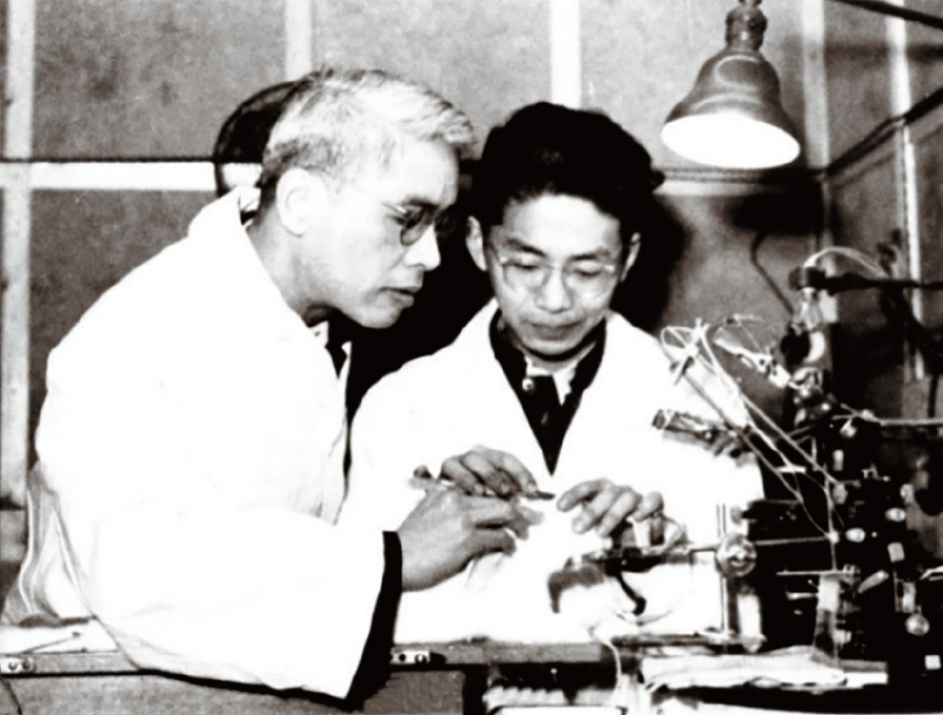
**Te-Pei Feng (left) instructed graduate students to conduct anatomy experiments on the rabbit brain** (Photo credit: Academician Library of Chinese Academy of Sciences https://yswk.csdl.ac.cn/)

**Figure 5:**
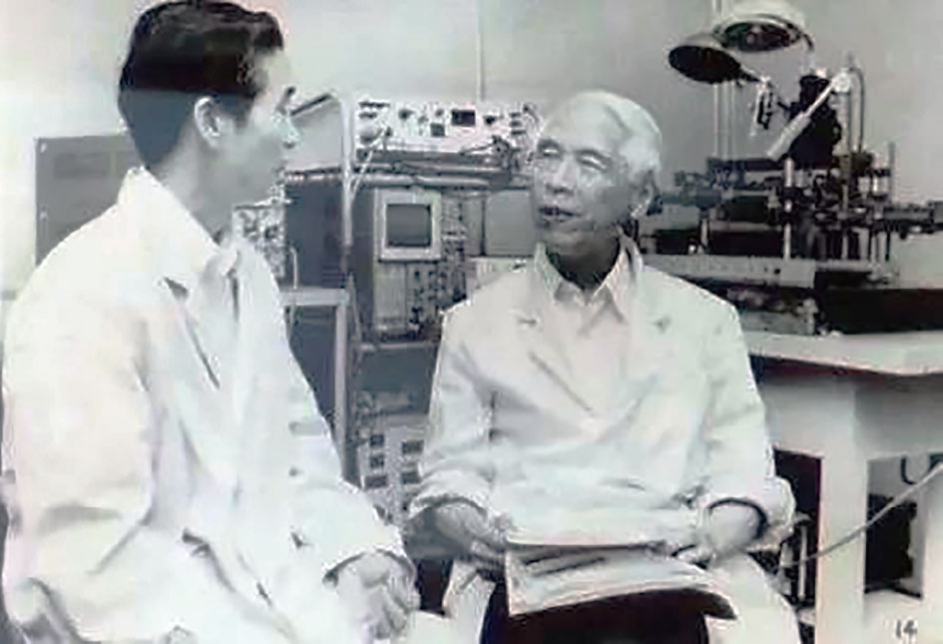
**Te-Pei Feng (right) still worked in the Laboratory in old age** (Photo credit: http://www.zhongdangwang.com/)

From 1936 to 1941, Feng published at least 26 papers in the *Chinese Journal of Physiology*. However, because of the Anti-Japanese War and the Pacific War, PUMC was forced to close and Feng had to leave in 1941, which interrupted his research. Among the crucial 26 papers, some of which are still cited today, is Feng’s pioneering work in the theory of chemical transmission at the neuromuscular junction, which led his laboratory to become a research center highly regarded by international peers (Figs. 4 and 5). In his autobiographical article “*Looking Back*, *Looking Forward*”, Feng described his main areas of research between 1936 and 1941: a. Accompanying the inhibition produced at the neuromuscular junction by high-frequency indirect stimulation (also generally called junctional inhibition), they found a local contraction surrounding the nerve endings (Feng and Yang, 1938; 1988); b. Calcium was shown to have various striking effects on the neuromuscular junction, which means that calcium causes each individual nerve impulse to liberate a larger or more concentrated amount of ACh from the nerve terminus; c. The post-tetanic potentiation (PTP) or facilitation of the endplate potential, which can last many minutes, was described for the first time (Feng, 1941); d. A new pre-junctional aspect of the eserine and curare effects, in addition to the post-tetanic effects, was thus brought to light, which is one of the first concrete examples of synaptic plasticity and is also the starting point of neuropharmacology (Feng, 1988).

**Figure 6:**
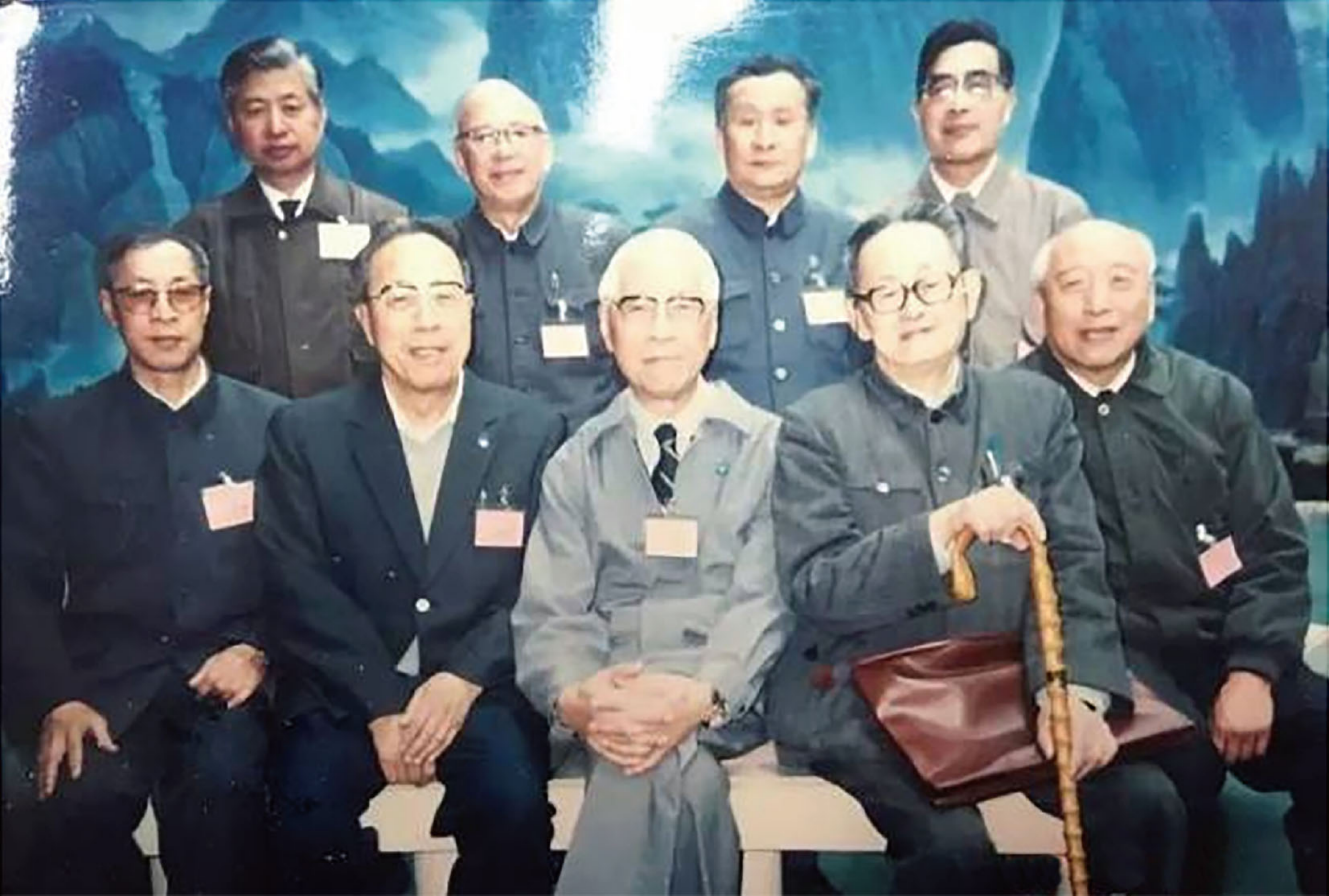
**In 1992, Te-Pei Feng (front row, in the middle) joined the 6th general assembly of the Chinese academy of sciences** (Photo credit: http://www.zhongdangwang.com/)

**Figure 7:**
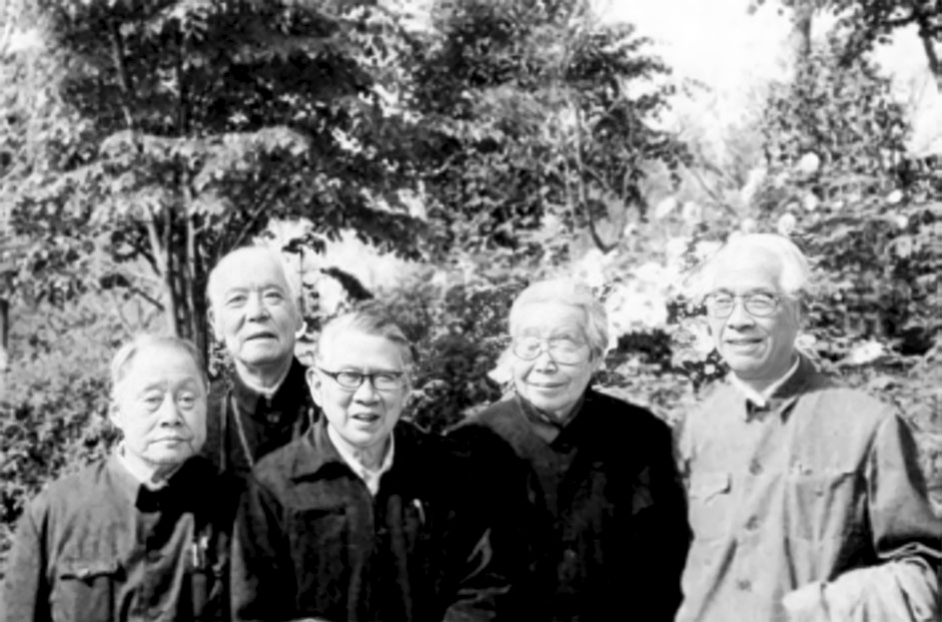
**In 1990, Te-Pei Feng (first from the right) and Hsiangtung Chang (second from the right) visited the Shanghai Botanical Garden** (Photo credit: Academician Library of Chinese Academy of Sciences https://yswk.csdl.ac.cn/)

In 1946, Feng visited the United States to buy experimental equipment and books for a new institute, and at the same time acquire new information at the scientific frontier. Afterwards, Feng worked at the Rockefeller Institute with Lorente de Nó for one year. However, Feng quickly returned to China due to an argument regarding the question whether the connective tissue sheath of frog or bull-frog nerve could act as a diffusion barrier. Lorente de Nó considered this idea utterly impossible (Lorente de Nó, 1950), vigorously challenging Feng’s opinion and scientific conclusions. However, Feng and Gerard had made a preliminary conclusion that the connective tissue sheath of nerves could act as a diffusion barrier as early as 1930 (Feng & Gerard, 1930). Thus, in the summer of 1947, Feng returned to China and re-verified this conclusion with the help of his assistant, Dr. Yumin Liu (刘育民). Finally, the experimental results corroborated the original conclusion of Feng and Gerard (Feng & Liu, 1949a, b; Feng & Liu, 1950). In 1948, Feng was selected as a member of Academia Sinica. Following the foundation of People’s Republic of China in 1949, Feng held multiple posts, and also made great strides in the study of peripheral nerves, central nerves and muscles (Fan, 2007). Feng was selected as a member of the Chinese Academy of Science in 1955 (Academician of People’s Republic of China) (Fig. 6). The following year, Feng went to Copenhagen and invited Hsiangtung Chang (张香桐) back to China to establish a neurophysiology research capacity with him (Fig. 7) (Qian, 1981; Zhang & Le, 2011). Chang came back to China in 1956 and set up the central nervous system research laboratory a few years later. In 1958, Feng joined the delegation of the Chinese Academy of Sciences to discuss the corporation with the Soviet Academy of Sciences. After returning from the USSR, Feng set up and directed the Qingdao working group, after which the first Chinese Marine Animal Physiology Laboratory was created in Qingdao in 1959 (Fan, 2007; 2011).

Feng studied nerve-muscle trophic relations in 1960. On the basis of his previous research experience, Feng considered that a new phenomenon should be discovered before launching a new project. Therefore, based on the known phenomenon of muscle atrophy following denervation, Feng led his assistants Xinwei Rong (荣辛未) and Wangyuan Wu (毋望远) to conduct experiments with high- and low-speed muscles, which have the most strikingly different contraction speeds among all muscles of chickens. Then, a surprising new phenomenon was discovered that the low contraction speed chicken muscle was strikingly hypertrophied. This discovery made an important contribution to elucidating the mechanism by which nerves determine the type of muscle fibers (Feng, Jung & Wu, 1962). Feng reported this new discovery in a muscle seminar held in Prague in 1962, and there is an academic consensus that this phenomenon was first discovered by him (The Editorial Department of *World Science*, 1985). In 1978, before Hill died, he wrote to Feng and told him that he is one of the two best students in his teaching career.

**Figure 8:**
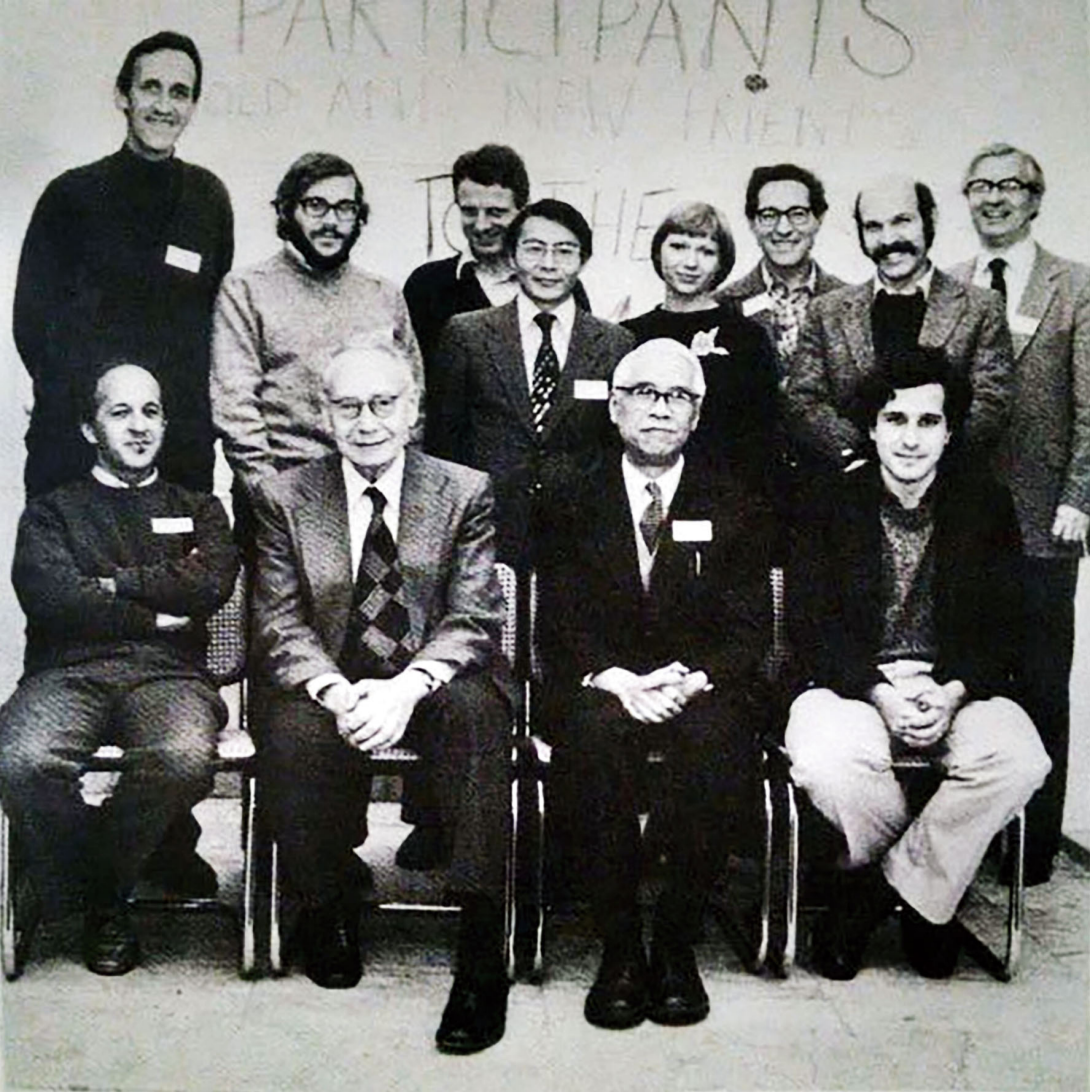
**In the 1980s, Te-Pei Feng (front row, third from the left) and Bernard Katz (front row, second from the left) attended international conferences** (Photo credit: http://www.zhongdangwang.com/)

**Figure 9:**
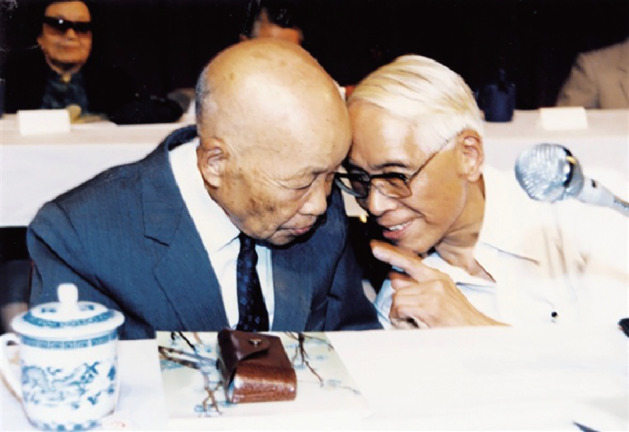
**In 1985, Te-Pei Feng (first from the right) had a cordial exchange with Buqing Su (苏步青) at the annual meeting of the European and American Alumni Association in Shanghai** (Photo credit: United Times 2017-3-6 http://cnepaper.com/)

**Figure 10:**
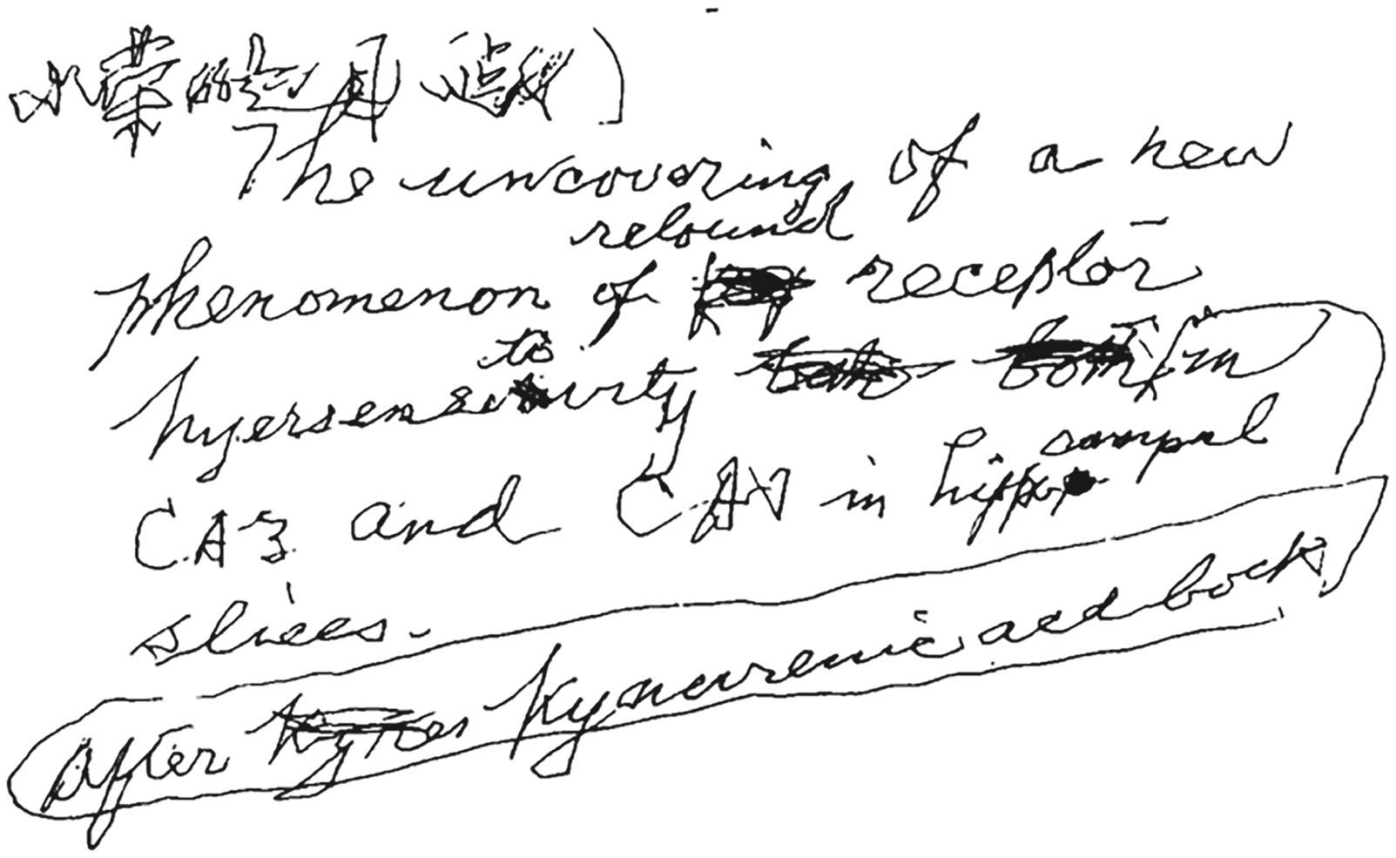
**On his hospital bed, Te-Pei Feng wrote down the last article title for a student**

Since the 1980s, Feng mainly explored the field of cellular and molecular synaptic physiology, focusing on synaptic plasticity in central synapses (Fig. 8). Feng devoted himself to the study of long-term potentiation (LTP) and made a significant contribution to the modern understanding of cellular mechanisms underlying LTP. In September 1985, Te-Pei Feng attended the annual meeting of the European and American Alumni Association in Shanghai (Fig. 9). In 1988, Feng wrote a retrospective of his whole scientific career in his biographic article *Looking Back*, *Looking Forward*, in response to an invitation by the *Annual Review of Neuroscience* (Feng, 1988). Even at 85 years old, Feng discovered a new phenomenon with his students, reporting in *PNAS* that the postsynaptic protein kinase C is essential for the induction and maintenance of long-term potentiation (LTP) (Wang & Feng, 1992; Feng, 1995). This was his first and last paper in *PNAS* since Feng became a foreign member of the National Academy of Sciences, United States in 1986. From 1983 to 1989, Feng was selected as an executive committee member of the International Union of Physiological Sciences (IUPS) for three consecutive sessions. In the last phase of his life, he underwent a tracheotomy and could not speak, but still summoned the strength to write down the title for his students’ latest research paper (Fig. 10) (Rong, 2007). On April 10th 1995, aged 88, Feng passed away in Shanghai.
